# Evaluation of vitamin D status, parathyroid hormone, and calcium among Iranian pregnant women with preeclampsia: A case-control study

**DOI:** 10.18502/ijrm.v17i10.5494

**Published:** 2019-11-28

**Authors:** Laaya Hamedanian, Bita Badehnoosh, Niloofar Razavi-Khorasani, Zinat Mohammadpour, Hassan Mozaffari-Khosravi

**Affiliations:** ^1^International Campus, Shahid Sadoughi University of Medical Sciences, Yazd, Iran.; ^2^Department of Gynecology and Obstetrics, School of Medicine, Alborz University of Medical Sciences, Karaj, Iran.; ^3^Liver Transplantation Research Center, Tehran University of Medical Sciences, Tehran, Iran.; ^4^Department of Nutrition, School of Public Health, Shahid Sadoughi University of Medical Sciences, Yazd, Iran.; ^5^Yazd Diabetes Research Center, Shahid Sadoughi University of Medical Sciences, Yazd, Iran.

**Keywords:** Preeclampsia, Vitamin D, 25-Hydroxyvitamin D, Pregnancy.

## Abstract

**Background:**

Preeclampsia is considered as a serious life-threatening condition that could affect both maternal and fetal outcome. Many studies have examined the association of nutritional factors with the incidence of preeclampsia. However, little is known about the possible role of vitamin D in the development of preeclampsia among the Iranian population.

**Objective:**

The aim of the present study was to evaluate the association between vitamin D status and preeclampsia

**Materials and Methods:**

A total of 120 pregnant women who were referred to Kamali and Alborz General Hospital located in the Karaj City were enrolled in this study and categorized into preeclamptic and control groups (n = 60/each). The clinical details of patients such as demographic characteristics and laboratory findings were obtained from the patients. The serum levels of vitamin D, calcium, phosphorus, and parathormone were also measured. Multivariate logistic regression analysis was used to assess for independent predictors of preeclampsia.

**Results:**

The mean age among pregnant women with preeclampsia and control group were 31.48 ± 5.25 and 29.01 ± 5.28, respectively. The mean body mass index among the preeclamptic group was 27.92 ± 4.98, which was significantly higher compared to the control group (p < 0.001). The serum vitamin D levels were significantly lower in women with preeclampsia compared to the control subjects (p = 0.007). Moreover, no correlation between vitamin D deficiency and predisposing factors of preeclampsia was observed after adjusting for confounding factors.

**Conclusion:**

Our study revealed that serum vitamin D level is significantly lower in among the pregnant women diagnosed with preeclampsia compared to the healthy subjects. However, no correlation was observed between the vitamin D status and the risk of preeclampsia development.

## 1. Introduction

Preeclampsia is a pregnancy disorder defined as elevated blood pressure and presence of proteinuria after the 20${}^{\mathrm{th}}$ wk of gestation that could affect various organ system in the body (1). This condition is estimated to occur in 2-8% of pregnancies and has been closely related to substantial maternal and fetal/neonatal morbidity and mortality (1, 2). Despite the increasing knowledge of its pathology and clinical manifestations, its exact underlying etiology remains largely unknown. However, several causative mechanisms have been identified to contribute to the development and progression of preeclampsia including placental origin, immunologic origin, and genetic predisposition factors (3, 4). Moreover, the main predisposing risk factors for developing preeclampsia include metabolic syndrome profile, endothelial dysfunction, oxidative stress, and increased inflammatory markers have also been suggested (5-7).

Vitamin D is a group of steroid hormones, derived from both diet and sunlight; it also plays an important role in innate immunity and many aspects of cellular functions (8). Vitamin D has been associated with a variety of pregnancy evolution such as gene regulation and expression in early placental development during pregnancy, fetomaternal immunological tolerance, and anti-inflammatory responses (9, 10). Vitamin D deficiency has recently been gaining attention in regards to its implication in the pathophysiology associated with preeclampsia (11). Previous studies have indicated that vitamin D improves endothelial function via regulating gene expression associated with placental invasion, and angiogenesis during normal implantation (12). Moreover, in-vitro and animal models have shown that vitamin D has a negative regulatory effect on the renin-angiotensin system by suppressing renin gene expression (13, 14). Although these findings have suggested that serum vitamin D levels are linked to blood pressure, yet it is largely unclear if vitamin D measurement should become part of routine hypertension prevention.

Since limited data exist regarding the association between serum vitamin D status and the presence of preeclampsia among Iranian women, the present study was conducted to compare vitamin D levels in two groups of pregnant women with preeclampsia and healthy subjects.

## 2. Materials and Methods

### Patients

The present prospective case-control study was conducted at two educational hospitals (Kamali and Alborz General Hospital) located in Karaj City from January 2016 to March 2017. A total of 60 pregnant women diagnosed with preeclampsia were assessed for eligibility based on the selection criteria. Patients were included if they were nulliparous preeclamptic women and aged 18-45 yr. Preeclampsia and proteinuria were defined according to the American College of Obstetricians and Gynecologists guidelines (15). Briefly, patients were considered to have preeclampsia if systolic blood pressure ≥ 140 mmHg and/or a diastolic pressure ≥ 90 mmHg. Proteinuria was defined as ≥ 300 mg/24 hr or 30 mg/dl (1+ dipstick) in at least one random urine sample after 20 wk of gestation. Patients with preexisting medical conditions such as the previous history of hypertension, diabetes, renal or liver failure, thyroid disease, malabsorption, multiple pregnancies, and history of intake of medications that may interfere with vitamin D or calcium metabolism such as anti-epileptics, anti-inflammatory agents, or antiretroviral drugs in the last six months were excluded. The control group consisted of 60 healthy nulliparous pregnant women who were age- and sex-matched with no history of major morbidity. The clinical details of patients such as demographic characteristics and laboratory findings were obtained from the patients.

### Measurements 

Blood samples were collected to evaluate vitamin D, serum calcium, and alkaline phosphatase levels. Vitamin D was measured using the ELISA method (Immune Diagnostic Systems, UK). A blood level of vitamin D above 30 ng/ml was considered sufficient, while values between 20 and 30 ng/ml as insufficient, and values below 20 ng/ml as deficient.

### Ethical consideration

Written informed consent was gathered from the subjects prior to sampling. The study was approved by the Ethics Committee of Shahid Sadoughi University of Medical Sciences (Code: IR.SSU.SPH.REC.1935.130). The process of this study was performed in accordance with the Declaration of Helsinki and other applicable guidelines, laws, and regulations (16).

### Statistical analysis 

Normally distributed data are represented as means and standard deviations. Non-normally distributed data are shown as medians with interquartile ranges. Kolmogorov-Smirnov test was used to evaluate the normal distribution of the quantitative variables. Statistical comparisons were performed using independent t-test (for quantitative variables) and the chi-square test (for qualitative variables). Multivariate logistic regression analysis was used to assess for independent predictors of preeclampsia. In addition, correlation analysis was used to evaluate the association between different continuous variables of the study. P < 0.05 was considered significant in all tests. All data were analyzed using SPSS 20.0 (Statistical Package for the Social Sciences, SPSS Inc.; Chicago, IL).

## 3. Results

The demographic characteristics of 60 women with preeclampsia and 60 control patients were summarized in Table I. The mean age at the time of study among pregnant women with preeclampsia and control group was 31.48 ± 5.25 and 29.01 ± 5.28, respectively. Women with preeclampsia had a significantly lower gestational age compared to the control group (p = 0.003). Among women with preeclampsia, 66.7% of subjects were found to be obese which was significantly higher than the control participants (p < 0.001). Positive family history of preeclampsia was present in 3 (5%) preeclamptic women. The mean BMI among the preeclamptic group was 27.92 ± 4.98, which was significantly higher compared to the control group (p < 0.001). The daily vitamin D supplementation of 1,000 IU was used among 12 patients with preeclampsia. The supplement usage such as calcium, Omega-3, iron, and vitamin D were significantly higher in women with preeclampsia compared to the control group. Moreover, there was no significant difference in regards to vitamin D deficiency across groups (p = 0.137). The serum vitamin D levels were significantly lower in women with preeclampsia compared to the control subjects (p = 0.007) (Table II).

**Figure 1:**
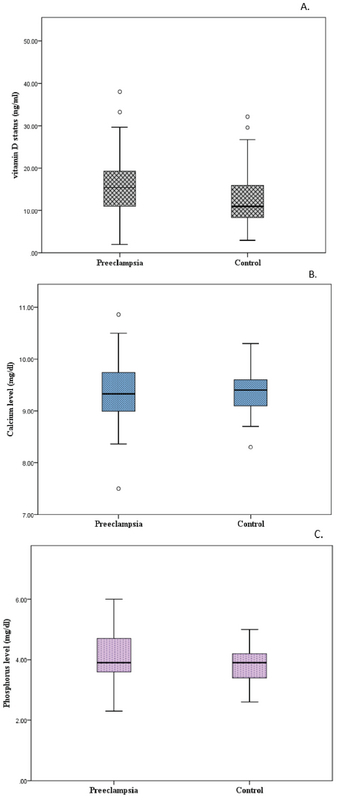
Comparison of different variables between women with preeclampsia and control group. (A) Differences in vitamin D levels; (B) Differences in Phosphorus levels; and (C) Differences in calcium levels.

The mean serum vitamin D levels were 6.88 ± 9.46 ng/ml, while in the control group, they were 13.41 ± 8.05 ng/ml. However, there was no significant difference in regards to serum phosphorus, calcium, and PTH levels between the two groups (Figure 1). The daily average dietary energy and nutrient intakes are summarized in Table III. The nutritional assessment revealed that women with preeclampsia had lower energy intake compared to the control subjects; however, this difference did not reach the statically significance (p = 0.688). Protein and fat intake were lower in the preeclamptic women than the control pregnant subjects, but the difference was not statistically significant (p > 0.05). After adjusting for all known potential confounding factors, multiple logistic regression was performed in order to determine the association between vitamin D deficiency and factors associated with preeclampsia development. The results suggest that there was no correlation between vitamin D deficiency and predisposing factors of preeclampsia (Table IV).

**Table 1:** Comparison of demographic characteristics between patients with preeclampsia and control group


**Parameters**	**Preeclampsia**	**Control**	**P-value**
Age (yr)*	31.48 ± 5.25	29.01 ± 5.28	0.017${}^{a}$
Gestational age (wk)*	227.86 ± 30.47	241.28 ± 33.37	0.003${}^{a}$
Height (cm)*	161.93 ± 5.94	162.31 ± 6.20	0.95${}^{a}$
BMI (kg/m${}^{2}$)*	27.92 ± 4.98	24.33 ± 3.87	< 0.001${}^{a}$
Weight before pregnancy (kg)*	73.26 ± 13.97	66.01 ± 10.30	< 0.001${}^{a}$
Weight at first trimester (kg)*	84.11 ± 13.92	76.23 ± 9.72	< 0.001${}^{a}$
Systolic blood pressure (mmHg)*	146.78 ± 7.84	105.51 ± 10.32	< 0.001${}^{a}$
Diastolic blood pressure (mmHg)*	55.60 ± 40.19	86.33 ± 82.21	0.268${}^{a}$
Educational level**		0.002${}^{b}$
Primary school	25 (41.6)	7 (11.6)	
Secondary school	27 (45)	35 (58.3)	
College	8 (13.3)	18 (30)	
History of miscarriage**	23 (38.3)	9 (15.1)	0.004${}^{b}$
History of preeclampsia**	3 (5)	0 (0)	0.244${}^{b}$
Family history of preeclampsia**	5 (8.3)	0 (0)	0.057${}^{b}$
Supplement intake**	48 (80.1)	32 (53.3)	0.002${}^{b}$
Calcium intake**	27 (45)	15 (25)	0.022
Omega-3 intake**	8 (13.3)	1 (1.7)	0.032
Iron supplement intake**	41 (68.3)	22 (36.7)	0.001
Vitamin D supplementation**	12 (20)	5 (8.3)	0.067
Vitamin D status**	13 (21.7)	23 (38.3)	0.137${}^{b}$
Sufficient**	4 (6.7)	3 (5)	
Insufficient**	43 (71.7)	34 (56.7)	
Deficiency**	13 (21.7)	23 (38.3)	
yr: Year; wk: Week; BMI: Body mass index			
Data presented as Mean ± SD; **Data presented as n (%)
${}^{c}$p < 0.05 was considered to be significant; ${}^{a}$Independent *t*-test; ${}^{b}$Chi-square test

**Table 2:** Comparison of serum levels of vitamin D, calcium, phosphorus, and PTH between the two groups


**Parameters**	**Preeclampsia**	**Control**	**P-value**
Serum vitamin D levels (ng/ml)	6.12 (4.57-11.21)	15.18 (7.67-18.83)	0.007${}^{a}$
Phosphorus (mg/dl)	4.08 ± 0.84	3.92 ± 0.71	0.204${}^{b}$
Calcium (mg/dl)	9.37 ± 0.61	9.37 ± 0.39	0.942${}^{b}$
Serum PTH (pg/ml)	23.05 (10.34-24.19)	24.56 (19.68-31.21)	0.956${}^{a}$
Data presented as Mean ± SD			
p < 0.05 was considered to be significant
${}^{a}$Mann-Whitney U-Test; ${}^{b}$Independent *t*-test

**Table 3:** Comparison of daily dietary energy and nutrient intakes between cases and controls


**Energy and nutrients**	**Preeclampsia**	**Control**	**P-value ${}^{a}$**
Energy consumption from protein (%)	17.40 ± 1.71	12.24 ± 2.01	0.818
Energy consumption from carbohydrate (%)	70.64 ± 5.04	70.37 ± 5.68	0.763
Energy consumption from fat (%)	11.93 ± 4.28	12.36 ± 4.77	0.871
Total protein (g)	142.93 ± 82.21	154.88 ±103.82	0.739
Total carbohydrate (g)	569.45 ± 302.51	653.47 ± 634.41	0.747
Total fat (g)	105.64 ± 84.21	121.25 ± 101.25	0.558
Energy (kcal)	3,692.36 ± 21.51	4,172.46 ± 32.84	0.688
Data presented as Mean ± SD	
p < 0.05 was considered to be significant
${}^{a}$Independent t-test

**Table 4:** Multivariate logistic regression analysis to determine the association between vitamin D deficiency and factors related to preeclampsia development


**Variables **	β	**Odds ratio (95% CI)**	**P-value ${}^{a}$**
Age	-0.013	0.98 (0.97-1.11)	0.07
Gestational age	0.067	1.06 (0.98-1.16)	0.11
Weight before pregnancy	0.029	1.02 (0.93-1.13)	0.55
Increased weight in first trimester	0.04	1.04 (0.94-1.15)	0.43
Vitamin D deficiency	-0.306	0.27 (0.04-1.66)	0.15
Vitamin D insufficiency	-0.053	0.55 (0.09-3.14)	0.51
p < 0.05 was considered to be significant			
${}^{a}$Wilcoxon-Mann-Whitney test

## 4. Discussion

In this study, we explored the differences in the vitamin D status between the two groups of pregnant women with preeclampsia and healthy subjects. Our results suggest that vitamin D levels in the preeclamptic pregnant subjects were significantly lower than the healthy ones. However, we did not find a correlation between vitamin D deficiency and predisposing factors associated with preeclampsia after adjusting for confounding factors using multivariate regression analysis. These observations are inconsistent with previous surveys, showing vitamin D insufficiency to be higher among preeclamptic pregnant women (17-19). Many hypotheses have been suggested in regards to the factors that could play a role in the pathogenesis of preeclampsia. In addition, vitamin D deficiency has been implicated as one of the factors in the etiology of preeclampsia, which stimulates systemic and vascular inflammation and results in inadequate placental development (20, 21). However, establishing a clear relationship between preeclampsia and vitamin D deficiency among pregnant women is difficult due to the complex nature of preeclampsia pathogenesis.

Low levels of serum vitamin D among preeclamptic women were observed in several studies. In a study done by Powe and colleagues (22), pregnant women who had serum vitamin D < 15 ng/ml were at an increased risk of development of preeclampsia. Similarly, Achkar and colleagues (18) reported that the risk of preeclampsia in women with vitamin D concentrations under 12 g/ml is higher. Although we observed that vitamin D deficiency was significantly more prevalent in our preeclamptic women, we could not find a causal relationship between the low levels of vitamin D concentration and increased risk of developing preeclampsia. These discrepancies in results from different surveys may be explained by the role of unmeasured confounding factors. Since most of the studies that were in favor of the correlation between vitamin D deficiency and preeclampsia, we did not assess genetic factors or other lifestyle variables that could potentially have an effect on serum vitamin D levels.

Previous observational studies reported that the risk of preeclampsia development may increase in women with higher weight gain prior to pregnancy (23, 24). In a study done by Tsai and colleagues, (25), it was reported that a higher rate of preeclampsia occurrence was commonly seen in pregnant women with excessive weight gain during pregnancy, whereas obese women with poor weight gain did not show similar results. Similarly, the current study showed that the BMI in the preeclamptic women was significantly higher compared to the control group. In contrast to the previous studies, we did not find a correlation between BMI and serum vitamin D levels after adjustment for confounding factors. Moreover, BMI was not considered as an independently predictable factor of vitamin D deficiency based on multivariable logistic regression. These findings could be explained by the lower mean gestational age in the preeclamptic women than in the control group. It has been suggested that there is an inverse relationship between serum vitamin D level and serum calcium and albumin level (26). However, we did not identify any association between these variables. This finding could be partly explained by the fact that vitamin D level is dependent on the exposure of sunlight and dietary factors and thereby little or no change in the level of vitamin D during pregnancy is expected.

Over the years, considerable attention has been directed to the role of malnutrition as a possible risk factor for developing preeclampsia (27). Lifestyle factors including increased and reduced dietary patterns of protein, fats or carbohydrates, and sodium could play an important role in the etiology of preeclampsia (28). Kazemian and colleagues (29) reported that higher energy intake was significantly associated with the risk of preeclampsia. Similarly, Clausen and colleagues (30) suggested that women with high intake of polyunsaturated fatty acids are associated with an increased likelihood of preeclampsia development. On the contrary, we did not find any difference in regards to any micro/macronutrients intake among either of the two groups. The main limitation of this study was the small sample size of the study. It should be noted that the other causes of vitamin D deficiency such as alteration in vitamin D metabolism during critical illness cannot be ruled out. Further well-designed randomized control trials with large sample size are warranted in order to elucidate the exact therapeutic effect of vitamin D supplementation in pregnant women with preeclampsia.

## 5. Conclusion

Our study revealed that serum vitamin D level is significantly lower in pregnant women diagnosed with preeclampsia compared to healthy subjects. Although the exact role of vitamin D in developing preeclampsia has not been fully elucidated, it may have a clinical implication in the management of these patients. However, no correlation was observed between the vitamin D status and the risk of preeclampsia development. Thus, it is unclear whether vitamin D supplementation in pregnant women may have a protective effect against preeclampsia, and needs to be examined in future well-designed clinical trials.

##  Conflict of Interest

The authors have no conflict of interest to declare.
